# The Value of Pharmacogenomics for White and Indigenous Americans after Kidney Transplantation

**DOI:** 10.3390/pharmacy11040125

**Published:** 2023-08-08

**Authors:** Alexandra Brady, Suman Misra, Mina Abdelmalek, Adrijana Kekic, Katie Kunze, Elisabeth Lim, Nicholas Jakob, Girish Mour, Mira T. Keddis

**Affiliations:** 1 Department of Nephrology and Hypertension, Mayo Clinic, Scottsdale, AZ 85259, USA; 2 Department of Pharmacy Clinical Practice, Mayo Clinic, Scottsdale, AZ 85259, USA; 3 Department of Statistics, Mayo Clinic, Scottsdale, AZ 85259, USA

**Keywords:** drug-metabolizing enzymes, genotype, Indigenous population, kidney transplantation, pharmacogenetics, pharmacogenomics, phenotype, polymorphism

## Abstract

Background: There is a paucity of evidence to inform the value of pharmacogenomic (PGx) results in patients after kidney transplant and how these results differ between Indigenous Americans and Whites. This study aims to identify the frequency of recommended medication changes based on PGx results and compare the pharmacogenomic (PGx) results and patients’ perceptions of the findings between a cohort of Indigenous American and White kidney transplant recipients. Methods: Thirty-one Indigenous Americans and fifty White kidney transplant recipients were studied prospectively. Genetic variants were identified using the OneOme RightMed PGx test of 27 genes. PGx pharmacist generated a report of the genetic variation and recommended changes. Pre- and post-qualitative patient surveys were obtained. Results: White and Indigenous American subjects had a similar mean number of medications at the time of PGx testing (mean 13 (SD 4.5)). In the entire cohort, 53% received beta blockers, 30% received antidepressants, 16% anticoagulation, 47% pain medication, and 25% statin therapy. Drug–gene interactions that warranted a clinical action were present in 21.5% of patients. In 12.7%, monitoring was recommended. Compared to the Whites, the Indigenous American patients had more normal CYP2C19 (*p* = 0.012) and CYP2D6 (*p* = 0.012) activities. The Indigenous American patients had more normal CYP4F2 (*p* = 0.004) and lower VKORC (*p* = 0.041) activities, phenotypes for warfarin drug dosing, and efficacy compared to the Whites. SLC6A4, which affects antidepressant metabolism, showed statistical differences between the two cohorts (*p* = 0.017); specifically, SLC6A4 had reduced expression in 45% of the Indigenous American patients compared to 20% of the White patients. There was no significant difference in patient perception before and after PGx. Conclusions: Kidney transplant recipients had several drug–gene interactions that were clinically actionable; over one-third of patients were likely to benefit from changes in medications or drug doses based on the PGx results. The Indigenous American patients differed in the expression of drug-metabolizing enzymes and drug transporters from the White patients.

## 1. Introduction

Pharmacogenomic (PGx) studies of genetic polymorphisms in drug-metabolizing enzymes, transporters, receptors, and drug targets may help to explain inter-individual variations in the drug efficacy and toxicity [1,2,3,4]. This information can provide significant insights to guide drug dosing and identify patients at risk of adverse drug reactions or a lack of drug efficacy. Patients with end-stage kidney disease transitioning to transplant are introduced to several new medications with potential drug–drug and drug–gene effects that may impact patient care [5,6,7].

The current standard of care for kidney transplant recipients includes regular laboratory monitoring of the immunosuppression levels to meet the narrow therapeutic index required to prevent acute rejection and over-exposure-related toxicities. Inter-individual variability in crucial drug-metabolizing enzymes has been reported across different races, highlighting the need for a more individualized approach to medication management post-transplant [8,9,10].

While several studies have explored the pharmacokinetics and pharmacodynamics of calcineurin inhibitors and have shown variable results on how PGx-directed dosing impacts clinical outcomes [11,12], to the best of our knowledge, there have not been any studies exploring how the PGx results may inform general medication management in patients after kidney transplant. Multiple studies showed an average number of prescribed medications for patients with end-stage renal disease between 10–12 [13,14,15]. Polypharmacy increases the likelihood of drug–drug interactions that may impact transplant and non-transplant medications. 

Patients with CYP3A5 *1/*1 or *1/*3 require up to 50% higher doses of tacrolimus to reach the target trough blood levels, while patients with CYP3A5 *3/*3 require lower doses [16]. It is known that there are significant ethnic differences in CYP3A5 polymorphisms [17,18,19,20,21]. Indigenous American patients represent a minority of kidney transplant recipients. Compared to Whites, they are especially prone to end-stage renal disease, but few PGx studies of Indigenous populations have been conducted [22,23,24]. Only one pharmacokinetic study of tacrolimus in 24 Indigenous American vs. 24 White subjects has been described to date [25,26]; CYP3A5 *3/*3 was more common in Indigenous Americans (88 versus 83% in Whites but not statistically significant) [25].

While there has been an increase in the use of PGx in transplant patients, patients from minoritized groups still need to be represented in these studies [22,24]. We sought to use our sizable transplant population pool at the Mayo Clinic in Arizona to identify the frequency of recommended medication changes based on PGx results and compare the pharmacogenomic (PGx) results and patients’ perceptions of the findings between a cohort of Indigenous American and White kidney transplant recipients.

## 2. Materials and Methods

The Mayo Clinic Institutional Review Board approved this study (IRB 10429-010). The Mayo Clinic Center for Individualized Medicine supported and funded this study. Our prospective cohort included 81 kidney transplant recipients with end-stage renal disease who were evaluated during the waitlisting period or shortly after receiving a kidney transplant. Patients were recruited between 2017–2021 at the time of annual wait-list evaluation at Mayo Clinic Arizona for kidney transplantation or during the first three months post-transplant surgery. Patients in the Indigenous American cohort were self-reported as American Indian/Alaskan Native in the medical record.

Genotype testing was performed through patient-directed buccal scraping. Samples were mailed to OneOme RightMed Test (Minneapolis, MN, USA) for PGx testing. Genotyping was conducted using a polymerase chain reaction (PCR) primer panel. Twenty-seven genes were analyzed. The results were made available in two weeks and reviewed by pharmacists with training in PGx. The pharmacist reviewed the results and determined the clinical recommendations based on the patient’s list of current medications at the time of the review. Links to the resources utilized by the pharmacist to guide the clinical recommendations are included in Supplemental Table S1. Based on each genetic profile, dosing recommendations of current medications were reported electronically. The pharmacist evaluation and final recommendations were communicated to the ordering provider (M.K.), who forwarded this information to the patient and the patient’s primary nephrologist via mail.

The categories of drug–gene interactions were based on the pharmacist’s review of the OneOme Right-Med Test results and resources outlined in Supplemental Table S1. The pharmacist reviewed the PGx results but applied clinical recommendations based on the patient’s prescribed medications at the time of the PGx test. There were three main categories: 1. clinically actionable; 2. monitoring required; 3. continue with current dosing. The clinically actionable category was based on drug–gene interactions with level A and A/B evidence according to CPIC and Pharmacogenomics knowledgebase level of evidence 1A (Supplemental Table S1). The monitoring required category applied to drug–gene interactions that had moderate or major drug–gene interactions identified based on the OneOme RightMed test report but lacked the level of evidence to support the guideline-directed clinical recommendation. The third category was labeled “continue with current dosing”, This category applied to the absence of drug–gene interactions concerning current medications.

Patients were asked to complete a Satisfaction Questionnaire before and after receiving their PGx results and pharmacist recommendations. Clinical data were obtained, including demographics, baseline comorbidities, and medications used.

The clinical data were analyzed using descriptive statistics and formal statistical tests for group differences. Categorical variables were compared using Chi-square tests with Fisher’s Exact tests used where appropriate. Kruskal-Wallis tests were used for comparisons between continuous variables. The rates of the categorical variables are reported. Median values and interquartile ranges are reported for continuous variables. Survey responses were compared across the two data collection time points by calculating descriptive statistics and Fisher’s Exact tests to explore differences in the response rates.

## 3. Results

In total, 81 (31 Indigenous Americans and 50 White) kidney transplant recipients underwent PGx testing between 2017 and 2021. The descriptive statistics are presented in Table 1. The mean age of the patients studied was 52.2 (SD 14.3) years; 40 (49.4%) were female. The Indigenous patients were younger (47.0 (SD 11.6) vs. 55.5 (SD 15.0), *p* = 0.010), more female (64.5% vs. 40.0%, *p* = 0.032), and had a higher rate of history of diabetes (58.1% vs. 18.4%, *p* < 0.001) than the White patients. Most patients in both groups required dialysis before transplant (87.1% of Indigenous American patients and 70.0% of White patients) and had hypertension (83.9% and 88.0%). There was no significant difference between the patient groups in terms of coronary artery disease, heart failure, peripheral arterial disease, and stroke.

The Indigenous American and White subjects showed a similar average number of medications at the time of PGx testing (Median = 12; IQR = 10.0, 16.0). In the entire cohort, 44 (53.1%) were taking beta-blockers, 24 (29.6%) were on antidepressants, 13 (16.0%) on anticoagulants, 38 (46.9%) on pain medications, and 20 (24.7%) on statins. There were no significant differences between the Indigenous Americans and Whites for the medications described. 

Table 2 highlights the phenotypes of the PGx panel showing the differences in phenotypic expression between the Indigenous Americans and White patients. Compared to the Whites, the Indigenous Americans had more normal CYP2C19 (71.0% vs. 38%, *p* < 0.05) and CYP2D6 (71.0% vs 44%, *p* < 0.05) activities. The Whites had more interindividual variation in the two metabolic enzymes, with higher rates of non-normal metabolizers. For example, in CYP2C19, more Whites ranged from ultrarapid (4% vs. 0.0%), rapid (30.0% vs. 6.5%), to poor metabolizers (4% vs. 0.0%) than Indigenous Americans. The genotypes that correspond to the phenotypes presented in Table 2 are included in Supplemental Table S2, which highlights several commonly found allelic combinations for CYP genes and other genes evaluated using the OneOme Right-Med Test.

There was a significant difference between the Indigenous American and White cohorts in the CYP4F2 and VKORC1 enzymes, which are critical for warfarin drug dosing and efficacy. The Indigenous American patients had a higher rate of normal versus low levels of CYP4F2 compared to the White patients (83.9% vs. 52.0%, *p* = 0.004). The Indigenous American subjects had lower VKORC1 activity than the White subjects (46.7% vs. 20.0%, *p* = 0.041). 

SLC6A4, which encodes the serotonin reuptake transporter, differed between the two cohorts. Higher rates of reduced expression (S/S genotype) were found in 45.2% of the Indigenous American patients compared to 20.0% of the White patients (*p* < 0.05), suggesting a decreased response to some selective serotonin reuptake inhibitors when compared to individuals with typical to increased expression (L/L genotype). There was a statistically significant difference in the catechol-O-methyltransferase (COMT) genotype, which influences pain response, with Indigenous Americans showing mostly high COMT activity (54.5%). In contrast, the Whites had intermediate (42.0%) activity (*p* < 0.05). Six (12%) White patients had an increased F2 phenotype, which is associated with an increased risk of thrombosis associated with prothrombin thrombophilia, compared to 1 Indigenous American (3.2%), *p* = 0.172. 

**Figure 1:**
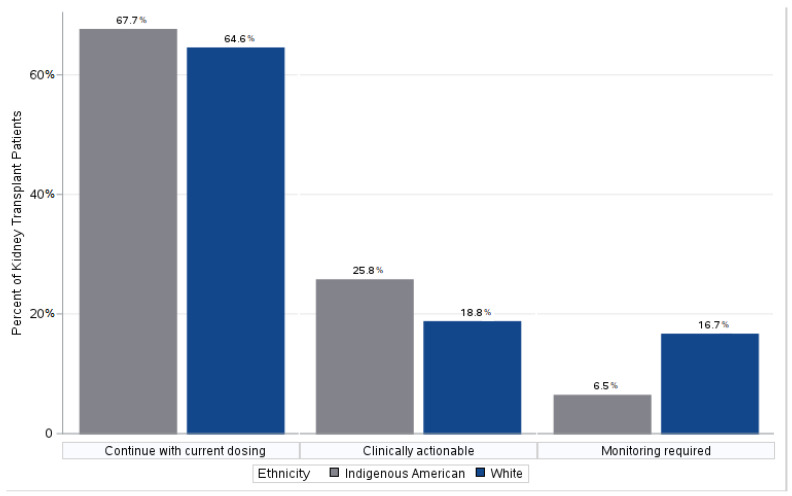
Percentage of patients with medical recommendations based on PGx testing between the two groups.

Overall, 52 patients did not have a drug–gene interaction based on their current medications. For 21.5% of the patients, the PGx pharmacist provided medication recommendations, and 12.7% were recommended monitoring for clinical efficacy or side effects, as shown in Figure 1. No difference was seen between the two cohorts in the number of recommendations for current medications (*p* = 0.8838)or drug–gene interactions (*p* = 0.3648). Table 3 highlights the drug–gene interactions that warranted a clinically actionable recommendation. 

A total of 59 of the subjects completed the pre-PGx Satisfaction Questionnaire, and 42 completed the post-PGx Satisfaction Questionnaire. There was no significant change in several items on the questionnaire between the pre-transplant and post-transplant scores, as shown in Supplemental Table S2. 

Analyzing the post-transplant survey responses split by the drug–gene interactions (Supplemental Table S3), there were very few differences in the rates across those who had clinically actionable (N = 5), minimally actionable (N = 7), or no interactions (N = 28). Of those who discussed with their transplant provider, few reported having any medication changes made (N = 6). Supplemental Table S4 shows the comparisons across the types of medication recommendations made, with most respondents (N = 28) having been recommended to continue with their current dose, six participants being referred for dose adjustment, and six participants told to use caution with their current medications.

## 4. Discussion

This study describes the phenotypic variations and relevant drug–gene interactions in 27 genes related to drug metabolism and transport in Indigenous American and White patients around the time of kidney transplant. PGx pharmacists provided medication recommendations in 21.5% of the cohort studied. This study also confirmed the known differences in multiple drug-metabolizing enzymes and drug transporters between Indigenous American compared to White patients, specifically in CYP2C19, CYP2D6, CYP4F2, VKORC1, SLC6A4, and COMT; some of these differences have clinical significance for drug dosing and clinical efficacy. 

Our findings are consistent with others that showed significant medication burden in patients with end-stage renal disease and those who have undergone transplantation [27,28,29,30,31]. We reported a median of 12 medications per patient. Similarly, a Dutch study with a larger sample size of 14,905 chronic kidney disease Stage G4/G5, 3872 dialysis patients and 8796 kidney transplant recipients reported a median number of 10, 12, and 11 medications for the respective groups [30]. Other studies in kidney transplant recipients reported a median number of medications that ranged between 6 and 22 at various time points post-transplant [27,28,31]. 

Our study showed that the PGx results revealed drug–gene interactions in one-third of the tested patients, of whom 22.5% would have benefited from medication recommendation and 12.7% from monitoring for clinical efficacy or side effects. We could not objectively assess the impact on patient care for patients who had these recommendations implemented versus those who did not. We also acknowledge that the implementation of PGx data must be in the context of the patient’s clinical care and consider other variables such as subjective medication tolerance, prior use, cost, and drug availability. Nonetheless, our findings show that PGx findings that warrant review, discussion, and medication adjustments were common in this cohort of patients. Future studies evaluating the impact of the clinical implementation of these changes may support the tangible clinical benefits of PGx testing.

Our study also highlighted PGx differences between the Indigenous and White Americans. The VKORC1 genotype explains 34% of the variability in the therapeutic warfarin dosing [32]. We showed that the Indigenous Americans had lower VKORC1 activity than the Whites, suggesting that Indigenous Americans are more sensitive to warfarin doses and may require lower doses. This finding is consistent with other studies showing a lower average warfarin dose requirement in Indigenous Americans compared to non-Indigenous Americans [33,34,35]. In our cohort of 81 patients, 5 were on warfarin (6%). Studies support that kidney transplant recipients benefit from lower warfarin doses than non-kidney transplant recipients, regardless of race, to achieve the therapeutic target [36]. We propose that PGx data may inform individualized warfarin dosing, particularly in Indigenous Americans, to support the therapeutic benefits and minimize bleeding complications. 

Prior studies on CYP2D6, CYP2C19, and CYP3A5 in Indigenous American populations showed similar allele frequencies compared to those observed in European Americans [37]. A systematic review [38] showed that Indigenous Americans had similar metabolization profiles of these enzymes to our study findings [38]. 

When comparing SLC6A4 phenotypes, Indigenous Americans were more likely to have reduced expression (S/S genotype) compared to Whites (45.2% vs. 20%, *p* < 0.05). Most of the Indigenous American (48.4%) and White populations (54.0%) had a typical to reduced expression (L/S or L/G genotype). A prior study found similar trends in serotonin transporter polymorphisms amongst White and Indigenous American populations [39]. They showed that 23% of Indigenous Americans had a reduced expression (S/S genotype), with the majority (51%) having a typical to reduced expression (L/S genotype). Reduced metabolism of SLC6A4 may contribute to a reduced treatment response with selective serotonin reuptake inhibitors.

While over one-third of the patients would have benefited from medication changes based on the PGx results, the patient perceptions did not differ significantly. This may be explained by significant delays in communicating the PGx test results with the referring providers, which ranged between 2 weeks and 6 months after the completion of the PGx test. Unique to our Transplant Center, patients are returned to their referring nephrologist. In this cohort, up to 80% of the patients were not followed at Mayo 4–8 months after their transplant but were referred to providers outside of Mayo, which limited the means of communication to faxed and mailed documents. We suspect the delay in communicating the results with the providers contributed to a lack of awareness of the results and perhaps a lower likelihood of implementing the recommended changes. Additionally, the patients did not meet with the pharmacist for medication therapy management, where there would have been further discussion on the PGx test results.

This study has several limitations that warrant discussion. First, the study used data on race and ethnicity that were self-reported by the patient from the electronic medical record. It did not differentiate White and Indigenous Americans based on geographic boundaries or genetic ancestry as recommended [40]. Second, this study used genotyping rather than sequencing methods, which may have missed other variants of significance, and was limited to the gene panel in the OneOme data, which may not have included other known genotypes involved in the drug metabolism of medications used in transplant. Third, the study was not designed to assess how these findings may impact clinical care or patient outcomes. The findings of this work should encourage future work to integrate pharmacogenomic testing in a standardized approach and ensure a more rapid result turnaround time and readily available PGx pharmacist guidance to improve patient and provider education. Lastly, the study is small, despite a long period of enrollment. Several unplanned factors contributed to low accrual despite a prolonged enrollment period: covid related delays, the disparity in access to transplants for Indigenous Americans, and the high turnover of research coordinator-support during covid.

## 5. Conclusions

Our study confirms that kidney transplant recipients require a high number of medications, and many of their non-transplant-related medications are influenced by phenotypic variances that are readily available in PGx. Our study sheds light on genetic variations in drug metabolism for Indigenous Americans, who are repeatedly underrepresented in drug studies. We found that more than one-third of kidney transplant patients may have an actionable, clinically relevant medication change based on PGx test results to allow for a more individualized pharmacological approach that maximizes drug efficacy while minimizing toxicity. A team approach that includes pharmacists with PGx expertise, patients, and physicians is needed to translate the novel and clinically relevant knowledge from PGx results into clinical practice. This will provide the cornerstone step to guide the measurable clinical impact of PGx integration in kidney transplant clinic practice.

## Figures and Tables

**Table 1:** Descriptive Statistic.

Descriptives				
	Ethnicity			
	White (N = 50)	Indigenous American (N = 31)	Total (N = 81)	*p*-Value
Gender, *n* (%)				0.032 ^1^
female	20 (40.0%)	20 (64.5%)	40 (49.4%)	
Age				0.010 ^2^
N	50	31	81	
Mean (SD)	55.5 (14.98)	47.0 (11.63)	52.2 (14.34)	
Beta blocker, *n* (%)				
	8 (16.0%)	14 (45.2%)	43 (53.1%)	
Antidepressant, *n* (%)				
	19 (38.0%)	5 (16.1%)	24 (29.6%)	
Anticoagulation, *n* (%)				
	11 (22.0%)	2 (6.45%)	13 (16.0%)	
Pain Medication, *n* (%)				
	24 (48.0%)	19 (61.3%)	43 (53.1%)	
Statin, *n* (%)				
	12 (24.0%)	8 25.8%)	20 (24.7%)	
Causes of end stage kidney disease, *n* (%)				
Diabetes (DM)	6 (12.0%)	15 (48.4%)	21 (25.9%)	
DM and glomerular disease	0 (0.0%)	1 (3.2%)	1 (1.2%)	
Hypertension	5 (10.0%)	2 (6.5%)	7 (8.6%)	
Polycystic kidney disease	8 (16.0%)	0 (0.0%)	8 (9.9%)	
Glomerular disease	16 (32.0%)	10 (32.3%)	26 (32.1%)	
Everything else	15 (30.0%)	3 (9.7%)	18 (22.2%)	
Dialysis, *n* (%)				0.078 ^1^
Yes	35 (70.0%)	27 (87.1%)	62 (76.5%)	
DM, *n* (%)				<0.001 ^1^
Yes	9 (18.4%)	18 (58.1%)	27 (33.8%)	
Hypertension, *n* (%)				0.598 ^1^
Yes	44 (88.0%)	26 (83.9%)	70 (86.4%)	
Hyperlipidemia, *n* (%)				0.221 ^1^
Yes	23 (46.0%)	10 (32.3%)	33 (40.7%)	
Coronary artery disease, *n* (%)				0.575 ^1^
Yes	3 (6.0%)	1 (3.2%)	4 (4.9%)	
Heart failure, *n* (%)				0.730 ^1^
Yes	1 (2.0%)	1 (3.2%)	2 (2.5%)	
Stroke, *n* (%)				0.165 ^1^
Yes	3 (6.0%)	0 (0.0%)	3 (3.7%)	
Peripheral arterial disease, *n* (%)				0.730 ^1^
Yes	1 (2.0%)	1 (3.2%)	2 (2.5%)	

^1^ Chi-Square *p*-value; ^2^ Kruskal-Wallis *p*-value.

**Table 2:** Frequency of actionable phenotypes by Indigenous Americans vs. White.

	Caucasian (N = 50)	Native American (N = 31)	Total (N = 81)	*p*-Value
CYP2C9 PHENOTYPE, *n* (%)				0.094 ^1^
Normal	31 (62.0%)	28 (90.3%)	59 (72.8%)	
Intermediate to normal	11 (22.0%)	2 (6.5%)	13 (16.0%)	
Intermediate	6 (12.0%)	1 (3.2%)	7 (8.6%)	
Poor to intermediate	1 (2.0%)	0 (0.0%)	1 (1.2%)	
Poor	1 (2.0%)	0 (0.0%)	1 (1.2%)	
CYP2C19 PHENOTYPE, *n* (%)				0.012 ^1^
Normal	19 (38.0%)	22 (71.0%)	41 (50.6%)	
Intermediate to normal	4 (8.0%)	0 (0.0%)	4 (4.9%)	
Intermediate	8 (16.0%)	7 (22.6%)	15 (18.5%)	
Poor	2 (4.0%)	0 (0.0%)	2 (2.5%)	
Rapid	15 (30.0%)	2 (6.5%)	17 (21.0%)	
Ultrarapid	2 (4.0%)	0 (0.0%)	2 (2.5%)	
CYP2D6 PHENOTYPE, *n* (%)				0.012 ^1^
Normal	22 (44.0%)	22 (71.0%)	44 (54.3%)	
Intermediate to normal	9 (18.0%)	0 (0.0%)	9 (11.1%)	
Intermediate	9 (18.0%)	8 (25.8%)	17 (21.0%)	
Poor to intermediate	3 (6.0%)	0 (0.0%)	3 (3.7%)	
Poor	7 (14.0%)	1 (3.2%)	8 (9.9%)	
CYP3A4 PHENOTYPE, *n* (%)				0.581 ^1^
Normal	45 (90.0%)	29 (93.5%)	74 (91.4%)	
Intermediate to normal	5 (10.0%)	2 (6.5%)	7 (8.6%)	
CYP3A5 PHENOTYPE, *n* (%)				0.207 ^1^
Intermediate	6 (12.0%)	7 (22.6%)	13 (16.0%)	
Poor	44 (88.0%)	24 (77.4%)	68 (84.0%)	
CYP4F2 PHENOTYPE, *n* (%)				0.004 ^1^
Normal	26 (52.0%)	26 (83.9%)	52 (64.2%)	
Reduced	24 (48.0%)	5 (16.1%)	29 (35.8%)	
COMT PHENOTYPE, *n* (%)				0.019 ^1^
Low activity	17 (34.0%)	6 (19.4%)	23 (28.4%)	
High activity	12 (24.0%)	17 (54.8%)	29 (35.8%)	
Intermediate	21 (42.0%)	8 (25.8%)	29 (35.8%)	
NUDT15 PHENOTYPE, *n* (%)				0.302 ^1^
Normal	49 (98.0%)	29 (93.5%)	78 (96.3%)	
Increased risk	1 (2.0%)	2 (6.5%)	3 (3.7%)	
SLC6A4 PHENOTYPE, *n* (%)				0.017 ^1^
Reduced	10 (20.0%)	14 (45.2%)	24 (29.6%)	
Typical to reduced	27 (54.0%)	15 (48.4%)	42 (51.9%)	
Typical To increased	13 (26.0%)	2 (6.5%)	15 (18.5%)	
SLCO1B1 PHENOTYPE, *n* (%)				0.434 ^1^
Normal	36 (72.0%)	21 (67.7%)	57 (70.4%)	
Decreased activity	14 (28.0%)	9 (29.0%)	23 (28.4%)	
Increased activity	0 (0.0%)	1 (3.2%)	1 (1.2%)	
TPMT PHENOTYPE, *n* (%)				0.157 ^1^
Normal	43 (86.0%)	24 (77.4%)	67 (82.7%)	
Intermediate Metabolizer	2 (4.0%)	5 (16.1%)	7 (8.6%)	
Increased	5 (10.0%)	2 (6.5%)	7 (8.6%)	
VKORC1 PHENOTYPE, *n* (%)				0.041 ^1^
Normal	9 (18.0%)	4 (13.3%)	13 (16.3%)	
Low activity	10 (20.0%)	14 (46.7%)	24 (30.0%)	
Intermediate	31 (62.0%)	12 (40.0%)	43 (53.8%)	
Missing	0	1	1	

^1^ Chi-Square *p*-value.

**Table 3:** List of drug–gene interactions that warranted warning to avoid certain medications or consideration for dose change based on CPIC guidelines (*n* = 17).

Patient	Gene	Drug	Clinical Recommendation Based on Current Medications
1-W	CYP2D6 ^a^ poor metabolizer	Oxycodone	-Avoid tramadol or codeine, be alert to symptoms of insufficient pain relief
2-W	CYP3A5*1/*3 ^b^ increased metabolism	Tacrolimus	-Increase starting dose 1.5 to 2 times recommended starting dose
3-IA	CYP3A5*1/*3 ^b^ increased metabolism	Tacrolimus	-Increase starting dose 1.5 to 2 times recommended starting dose
4-IA	CYP3A5 *1/*3 ^b^ increased metabolism	Tacrolimus	-Increase starting dose 1.5 to 2 times recommended starting dose
5-IA	CYP3A5 *1/*3 ^b^ increased metabolism	Tacrolimus	-Increase starting dose 1.5 to 2 times recommended starting dose
6-IA	CYP3A5 *1/*3 ^b^ increased metabolism	Tacrolimus	-Increase starting dose 1.5 to 2 times recommended starting dose
7-IA	CYP3A5 *1/*3 ^b^ increased metabolism	Tacrolimus	-Increase starting dose 1.5 to 2 times recommended starting dose
8-W	CYP3A5 *1/*3 ^b^ increased metabolism	Tacrolimus	-Increase starting dose 1.5 to 2 times recommended starting dose
9-W	CYP3A5 *1/*3 ^b^ increased metabolism	Tacrolimus	-Increase starting dose 1.5 to 2 times recommended starting dose
10-W	CYP2C19 ^c^ increased metabolism	Omeprazole Citalopram	-Consider dose increase by 100–200% -Consider an alternative drug
11-W	CYP3A5 *1/*3 ^b^ increased metabolism	Tacrolimus	-Increase starting dose 1.5 to 2 times recommended starting dose
12-W	CYP3A5 *1/*3 ^b^ increased metabolism	Tacrolimus	-Increase starting dose 1.5 to 2 times recommended starting dose
13-IA	CYP3A5 *1/*3 ^b^ increased metabolism	Tacrolimus	-Increase starting dose 1.5 to 2 times recommended starting dose
14-IA	CYP3A5 *1/*3 ^b^ increased metabolism	Tacrolimus	-Increase starting dose 1.5 to 2 times recommended starting dose
15-W	CYP3A5 *1/*3 ^b^ increased metabolism	Tacrolimus	-Increase starting dose 1.5 to 2 times recommended starting dose
16-W	-CYP2D6 poor to intermediate metabolizer -CYP3A4 intermediate metabolizer	Oxycodone	-Avoid tramadol or codeine
17-IA	CYP2D6 poor metabolizer	Oxycodone	-Avoid tramadol or codeine, be alert to symptoms of insufficient pain relief

^a^ https://cpicpgx.org/guidelines/guideline-for-selective-serotonin-reuptake-inhibitors-and-cyp2d6-and-cyp2c19/, https://cpicpgx.org/guidelines/guideline-for-codeine-and-cyp2d6/ (accessed on 1 December 2022). ^b^ https://cpicpgx.org/guidelines/guideline-for-tacrolimus-and-cyp3a5/ (accessed on 1 December 2022). ^c^ https://cpicpgx.org/guidelines/guideline-for-selective-serotonin-reuptake-inhibitors-and-cyp2d6-and-cyp2c19/, https://cpicpgx.org/guidelines/cpic-guideline-for-proton-pump-inhibitors-and-cyp2c19/ (accessed on 1 December 2022).

## Data Availability

Deidentified data presented in this study are available on request from the corresponding author. The data are not publicly available due to privacy and ethical reasons, as per the IRB.

## Supplementary Materials

The following supporting information can be downloaded at: https://www.mdpi.com/article/10.3390/pharmacy11040125/s1, Table S1: Resources utilized by pharmacogenomics pharmacists to guide clinical recommendations. Table S2: A list of categories of phenotypes and corresponding genotypes used for this study. Table S3: Comparison of Survey Results for Patients Pre-transplant and Post-transplant, Table S4: Comparison of Responses to Post-transplant Survey by Observed Drug–Gene Interactions, Table S5: Comparison Responses to the Post-transplant Survey by Medication Recommendations.
