# Supplementary material for: The Value of Pharmacogenomics for White and Indigenous Americans after Kidney Transplantation
Source: Pharmacy (Basel). 2023 Aug 8;11(4):125. doi: 10.3390/pharmacy11040125 (PMC10457738; doi:10.3390/pharmacy11040125)
Supplement: Supplementary file 1 [file pharmacy-11-00125-s001.zip › pharmacy-2411640-supplementary.pdf]

pharmacy

MDPI

Supplementary Information

Table S1:

Resources utilized by pharmacogenomic pharmacist to guide clinical recommendations

**Clinical Pharmacogenetics Implementation Consortium (CPIC)**<br><br>**CPIC levels of evidence**	**https://cpicpgx.org/genes-drugs/**<br><br>**https://cpicpgx.org/prioritization/**
Up to Date	www.uptodate.com
Pharmacogenomics Knowledgebase	www.pharmgkb.org
Royal Dutch Pharmacists Association- Pharmacogenetics Working Group	https://www.pharmgkb.org/literature/7283333<br>https://www.pharmgkb.org/literature/8564161
OneOme RightMed Test	https://oneome.com/rightmed-test/

Table S2: Allelic combinations that correlate with the phenotypes presented in Table 2

**Phenotype**	**Genotype**
**CYP2C9 PHENOTYPE**, n (%)	
Normal	*1/*1
Intermediate to normal	*1/*2
Intermediate	*1/*3
Poor to intermediate	*2/*3
Poor	*3/*3
**CYP2C19 PHENOTYPE**, n (%)	
Normal	*1/*1
Intermediate to normal	*2/*17
Intermediate	*1/*2
Poor	*2/*2
Rapid	*1/*17
Ultrarapid	*17/*17
**CYP2D6 PHENOTYPE**, n (%)	
Normal	*1/*2A
Intermediate to normal	*1/*41
Intermediate	*2A/*4
Poor to intermediate	*13/*41
Poor	*4/*4
**CYP3A4 PHENOTYPE**, n (%)	
Normal	*1/*1
Intermediate to normal	*1/*22
**CYP3A5 PHENOTYPE**, n (%)	
Intermediate	*1/*3
Poor	*3/*3

**CYP4F2 PHENOTYPE**, n (%)	
Normal	*1/*1
Reduced	*1/*3
**COMT PHENOTYPE**, n (%)	
Low activity	rs4680 AA
High activity	rs4680 GG
Intermediate	rs4680 GA
**NUDT15 PHENOTYPE**, n (%)	
Normal	rs116855232 CC
Increased risk	rs116855232 CT
**SLC6A4 PHENOTYPE**, n (%)	
Reduced	(Sa/Sa)
Typical to reduced	(La/Sa) or (La/Lg)
Typical To increased	(La/La)
**SLCO1B1 PHENOTYPE**, n (%)	
Normal	*1A/*1B
Decreased activity	*1A/*5
Increased activity	*1A/*15 or *1B/*5
**TPMT PHENOTYPE**, n (%)	
Normal	*1/*1
Intermediate Metabolizer	*1/*3C
Increased	*1/*3A
**VKORC1 PHENOTYPE**, n (%)	
Normal	rs9923231 GG
Low activity	rs9923231 AA
Intermediate	rs9923231 GA

Table S3: Comparison of Survey Results for Patients Pre-transplant and Post-transplant

	Post (N=42)	Pre (N=59)	Total (N=101)	p value
**In general, would you say your health is:**				0.522[1]
N-Miss	3	1	4	
Excellent	4 (10.3%)	2 (3.4%)	6 (6.2%)	
Fair	5 (12.8%)	9 (15.5%)	14 (14.4%)	
Good	14 (35.9%)	28 (48.3%)	42 (43.3%)	
Poor	2 (5.1%)	3 (5.2%)	5 (5.2%)	
Very Good	14 (35.9%)	16 (27.6%)	30 (30.9%)	
**How important of a role do you think your genetics take in your kidney and transplant care?**				0.580[1]
N-Miss	1	1	2	
Important	10 (24.4%)	18 (31.0%)	28 (28.3%)	
Neutral	5 (12.2%)	7 (12.1%)	12 (12.1%)	
Not Important	2 (4.9%)	3 (5.2%)	5 (5.1%)	
Somewhat Important	5 (12.2%)	12 (20.7%)	17 (17.2%)	
Very Important	19 (46.3%)	18 (31.0%)	37 (37.4%)	
**How concerned are you that your test results might show that you need to switch to a different prescription drug?**				0.901[1]
N-Miss	6	3	9	
Extremely Concerned	5 (13.9%)	9 (16.1%)	14 (15.2%)	
Not At All Concerned	16 (44.4%)	24 (42.9%)	40 (43.5%)	
Quite Concerned	4 (11.1%)	9 (16.1%)	13 (14.1%)	
Slightly Concerned	11 (30.6%)	14 (25.0%)	25 (27.2%)	
**Knowing how my body may respond to certain prescription drugs would bring me peace of mind**				< 0.001[1]
N-Miss	2	1	3	
Agree	25 (62.5%)	17 (29.3%)	42 (42.9%)	
Disagree	1 (2.5%)	0 (0.0%)	1 (1.0%)	
Neither Agree Or Disagree	4 (10.0%)	2 (3.4%)	6 (6.1%)	
Strongly Agree	10 (25.0%)	39 (67.2%)	49 (50.0%)	
**Knowing how my body may respond to certain prescription drugs would give me more control over my health**				< 0.001[1]
N-Miss	2	2	4	
Agree	22 (55.0%)	17 (29.8%)	39 (40.2%)	
Disagree	1 (2.5%)	0 (0.0%)	1 (1.0%)	
Neither Agree Or Disagree	6 (15.0%)	0 (0.0%)	6 (6.2%)	
Strongly Agree	10 (25.0%)	40 (70.2%)	50 (51.5%)	
Strongly Disagree	1 (2.5%)	0 (0.0%)	1 (1.0%)	
**Knowing how my body may respond to certain prescription drugs would be useful to me immediately**				< 0.001[1]
N-Miss	3	2	5	
Agree	26 (66.7%)	16 (28.1%)	42 (43.8%)	
Disagree	1 (2.6%)	0 (0.0%)	1 (1.0%)	
Neither Agree Or Disagree	6 (15.4%)	5 (8.8%)	11 (11.5%)	
Strongly Agree	6 (15.4%)	36 (63.2%)	42 (43.8%)	
**Knowing how my body may respond to certain prescription drugs would be useful to me in the future**				0.029[1]
N-Miss	2	2	4	
Agree	22 (55.0%)	18 (31.6%)	40 (41.2%)	

Neither Agree Or Disagree	3 (7.5%)	2 (3.5%)	5 (5.2%)	
Strongly Agree	15 (37.5%)	37 (64.9%)	52 (53.6%)	

1. Fisher's Exact Test for Count Data

Table S4: Comparison of Responses to Post-transplant Survey by Observed Drug-Gene Interactions

	**Clinically Actionable (N=5)**	**Minimal (N=7)**	**None (N=28)**	**Total (N=40)**	**p value**
**In general, would you say your health is:**					0.546[1]
N-Miss	0	2	1	3	
Excellent	0 (0.0%)	0 (0.0%)	3 (11.1%)	3 (8.1%)	
Fair	2 (40.0%)	0 (0.0%)	3 (11.1%)	5 (13.5%)	
Good	2 (40.0%)	1 (20.0%)	11 (40.7%)	14 (37.8%)	
Poor	0 (0.0%)	0 (0.0%)	1 (3.7%)	1 (2.7%)	
Very Good	1 (20.0%)	4 (80.0%)	9 (33.3%)	14 (37.8%)	
**How important of a role do you think your genetics take in your kidney and transplant care?**					0.394[1]
N-Miss	0	1	0	1	
Important	2 (40.0%)	1 (16.7%)	6 (21.4%)	9 (23.1%)	
Neutral	0 (0.0%)	0 (0.0%)	5 (17.9%)	5 (12.8%)	
Not Important	0 (0.0%)	0 (0.0%)	2 (7.1%)	2 (5.1%)	
Somewhat Important	2 (40.0%)	0 (0.0%)	3 (10.7%)	5 (12.8%)	
Very Important	1 (20.0%)	5 (83.3%)	12 (42.9%)	18 (46.2%)	
**I have had a discussion with my primary care provider regarding my pharmacogenomics test results**					0.382[1]
N-Miss	0	0	1	1	
Don't Know	2 (40.0%)	1 (14.3%)	3 (11.1%)	6 (15.4%)	
No	3 (60.0%)	4 (57.1%)	20 (74.1%)	27 (69.2%)	
Yes	0 (0.0%)	2 (28.6%)	4 (14.8%)	6 (15.4%)	
**I have had a discussion with my kidney transplant provider regarding my pharmacogenomics test results**					0.460[1]
N-Miss	0	0	1	1	
Don't Know	0 (0.0%)	1 (14.3%)	3 (11.1%)	4 (10.3%)	
No	3 (60.0%)	3 (42.9%)	19 (70.4%)	25 (64.1%)	
Yes	2 (40.0%)	3 (42.9%)	5 (18.5%)	10 (25.6%)	
**If you answered yes to #3 OR #4, were there any changes made to any of your prescriptions?**					0.009[1]
N-Miss	3	3	20	26	
Don't Know	1 (50.0%)	0 (0.0%)	0 (0.0%)	1 (7.1%)	
No	1 (50.0%)	0 (0.0%)	6 (75.0%)	7 (50.0%)	
Yes	0 (0.0%)	4 (100.0%)	2 (25.0%)	6 (42.9%)	
**If changes were made to your medications, how satisfied are you with these changes?**					0.643[1]
N-Miss	3	3	19	25	
Extremely Dissatisfied	0 (0.0%)	0 (0.0%)	1 (11.1%)	1 (6.7%)	
Extremely Satisfied	0 (0.0%)	1 (25.0%)	4 (44.4%)	5 (33.3%)	
Satisfied	0 (0.0%)	2 (50.0%)	2 (22.2%)	4 (26.7%)	
Very Satisfied	2 (100.0%)	1 (25.0%)	2 (22.2%)	5 (33.3%)	
**If you have reviewed study results, how concerned are you that your results may indicate that you need a**					1.000[1]

**different dose of a prescription drug you are currently taking?**					
N-Miss	1	1	5	7	
Extremely Concerned	0 (0.0%)	1 (16.7%)	3 (13.0%)	4 (12.1%)	
Not At All Concerned	2 (50.0%)	3 (50.0%)	10 (43.5%)	15 (45.5%)	
Quite Concerned	0 (0.0%)	0 (0.0%)	1 (4.3%)	1 (3.0%)	
Slightly Concerned	2 (50.0%)	2 (33.3%)	9 (39.1%)	13 (39.4%)	
**How concerned are you that your test results might show that you need to switch to a different prescription drug?**					0.861 [1]
N-Miss	0	1	5	6	
Extremely Concerned	0 (0.0%)	1 (16.7%)	3 (13.0%)	4 (11.8%)	
Not At All Concerned	2 (40.0%)	4 (66.7%)	9 (39.1%)	15 (44.1%)	
Quite Concerned	1 (20.0%)	0 (0.0%)	3 (13.0%)	4 (11.8%)	
Slightly Concerned	2 (40.0%)	1 (16.7%)	8 (34.8%)	11 (32.4%)	
**Knowing how my body may respond to certain prescription drugs would bring me peace of mind**					0.198 [1]
N-Miss	0	1	1	2	
Agree	3 (60.0%)	2 (33.3%)	19 (70.4%)	24 (63.2%)	
Disagree	0 (0.0%)	0 (0.0%)	1 (3.7%)	1 (2.6%)	
Neither Agree Or Disagree	0 (0.0%)	0 (0.0%)	3 (11.1%)	3 (7.9%)	
Strongly Agree	2 (40.0%)	4 (66.7%)	4 (14.8%)	10 (26.3%)	
**Knowing how my body may respond to certain prescription drugs would be useful to me immediately**					0.546 [1]
N-Miss	0	1	2	3	
Agree	2 (40.0%)	5 (83.3%)	18 (69.2%)	25 (67.6%)	
Disagree	0 (0.0%)	0 (0.0%)	1 (3.8%)	1 (2.7%)	
Neither Agree Or Disagree	1 (20.0%)	1 (16.7%)	4 (15.4%)	6 (16.2%)	
Strongly Agree	2 (40.0%)	0 (0.0%)	3 (11.5%)	5 (13.5%)	
**Knowing how my body may respond to certain prescription drugs would give me more control over my health**					0.457 [1]
N-Miss	0	1	1	2	
Agree	1 (20.0%)	3 (50.0%)	17 (63.0%)	21 (55.3%)	
Disagree	0 (0.0%)	0 (0.0%)	1 (3.7%)	1 (2.6%)	
Neither Agree Or Disagree	1 (20.0%)	1 (16.7%)	4 (14.8%)	6 (15.8%)	
Strongly Agree	3 (60.0%)	2 (33.3%)	4 (14.8%)	9 (23.7%)	
Strongly Disagree	0 (0.0%)	0 (0.0%)	1 (3.7%)	1 (2.6%)	
**Knowing how my body may respond to certain prescription drugs would be useful to me in the future**					0.118 [1]
N-Miss	0	1	1	2	
Agree	2 (40.0%)	1 (16.7%)	18 (66.7%)	21 (55.3%)	
Neither Agree Or Disagree	0 (0.0%)	1 (16.7%)	2 (7.4%)	3 (7.9%)	
Strongly Agree	3 (60.0%)	4 (66.7%)	7 (25.9%)	14 (36.8%)	
**How valuable are your pharmacogenomics results?**					0.963 [1]
N-Miss	0	1	2	3	
Extremely Valuable	1 (20.0%)	1 (16.7%)	5 (19.2%)	7 (18.9%)	
Not At All Valuable	0 (0.0%)	0 (0.0%)	1 (3.8%)	1 (2.7%)	
Quite Valuable	3 (60.0%)	2 (33.3%)	12 (46.2%)	17 (45.9%)	
Slightly Valuable	1 (20.0%)	3 (50.0%)	8 (30.8%)	12 (32.4%)	

**Would you recommend you friends or family to have pharmacogenomics testing?**					0.161[1]
N-Miss	0	0	1	1	
Don't Know	3 (60.0%)	0 (0.0%)	6 (22.2%)	9 (23.1%)	
No	0 (0.0%)	0 (0.0%)	2 (7.4%)	2 (5.1%)	
Yes	2 (40.0%)	7 (100.0%)	19 (70.4%)	28 (71.8%)	

1. Fisher's Exact Test for Count Data

Table S5: Comparison Responses to the Post-transplant Survey by Medication Recommendations

	Continue With Current Dosing (N=28)	Dose Adjustment (N=6)	Use Caution (N=6)	Total (N=40)	p value
**In general, would you say your health is:**					0.761[1]
N-Miss	1	0	2	3	
Excellent	3 (11.1%)	0 (0.0%)	0 (0.0%)	3 (8.1%)	
Fair	3 (11.1%)	2 (33.3%)	0 (0.0%)	5 (13.5%)	
Good	11 (40.7%)	2 (33.3%)	1 (25.0%)	14 (37.8%)	
Poor	1 (3.7%)	0 (0.0%)	0 (0.0%)	1 (2.7%)	
Very Good	9 (33.3%)	2 (33.3%)	3 (75.0%)	14 (37.8%)	
**How important of a role do you think your genetics take in your kidney and transplant care?**					0.724[1]
N-Miss	0	0	1	1	
Important	6 (21.4%)	2 (33.3%)	1 (20.0%)	9 (23.1%)	
Neutral	5 (17.9%)	0 (0.0%)	0 (0.0%)	5 (12.8%)	
Not Important	2 (7.1%)	0 (0.0%)	0 (0.0%)	2 (5.1%)	
Somewhat Important	3 (10.7%)	2 (33.3%)	0 (0.0%)	5 (12.8%)	
Very Important	12 (42.9%)	2 (33.3%)	4 (80.0%)	18 (46.2%)	
**I have had a discussion with my primary care provider regarding my pharmacogenomics test results**					0.299[1]
N-Miss	1	0	0	1	
Don't Know	3 (11.1%)	2 (33.3%)	1 (16.7%)	6 (15.4%)	
No	20 (74.1%)	4 (66.7%)	3 (50.0%)	27 (69.2%)	
Yes	4 (14.8%)	0 (0.0%)	2 (33.3%)	6 (15.4%)	
**I have had a discussion with my kidney transplant provider regarding my pharmacogenomics test results**					0.489[1]
N-Miss	1	0	0	1	
Don't Know	3 (11.1%)	1 (16.7%)	0 (0.0%)	4 (10.3%)	
No	19 (70.4%)	3 (50.0%)	3 (50.0%)	25 (64.1%)	
Yes	5 (18.5%)	2 (33.3%)	3 (50.0%)	10 (25.6%)	
**If you answered yes to #3 OR #4, were there any changes made to any of your prescriptions?**					0.009[1]
N-Miss	20	4	2	26	
Don't Know	0 (0.0%)	1 (50.0%)	0 (0.0%)	1 (7.1%)	
No	6 (75.0%)	1 (50.0%)	0 (0.0%)	7 (50.0%)	
Yes	2 (25.0%)	0 (0.0%)	4 (100.0%)	6 (42.9%)	
**If changes were made to your medications, how satisfied are you with these changes?**					0.643[1]

N-Miss	19	4	2	25	
Extremely Dissatisfied	1 (11.1%)	0 (0.0%)	0 (0.0%)	1 (6.7%)	
Extremely Satisfied	4 (44.4%)	0 (0.0%)	1 (25.0%)	5 (33.3%)	
Satisfied	2 (22.2%)	0 (0.0%)	2 (50.0%)	4 (26.7%)	
Very Satisfied	2 (22.2%)	2 (100.0%)	1 (25.0%)	5 (33.3%)	
**If you have reviewed study results, how concerned are you that your results may indicate that you need a different dose of a prescription drug you are currently taking?**					1.000[1]
N-Miss	5	1	1	7	
Extremely Concerned	3 (13.0%)	0 (0.0%)	1 (20.0%)	4 (12.1%)	
Not At All Concerned	10 (43.5%)	3 (60.0%)	2 (40.0%)	15 (45.5%)	
Quite Concerned	1 (4.3%)	0 (0.0%)	0 (0.0%)	1 (3.0%)	
Slightly Concerned	9 (39.1%)	2 (40.0%)	2 (40.0%)	13 (39.4%)	
**How concerned are you that your test results might show that you need to switch to a different prescription drug?**					0.978[1]
N-Miss	5	0	1	6	
Extremely Concerned	3 (13.0%)	0 (0.0%)	1 (20.0%)	4 (11.8%)	
Not At All Concerned	9 (39.1%)	3 (50.0%)	3 (60.0%)	15 (44.1%)	
Quite Concerned	3 (13.0%)	1 (16.7%)	0 (0.0%)	4 (11.8%)	
Slightly Concerned	8 (34.8%)	2 (33.3%)	1 (20.0%)	11 (32.4%)	
**Knowing how my body may respond to certain prescription drugs would bring me peace of mind**					0.275[1]
N-Miss	1	0	1	2	
Agree	19 (70.4%)	3 (50.0%)	2 (40.0%)	24 (63.2%)	
Disagree	1 (3.7%)	0 (0.0%)	0 (0.0%)	1 (2.6%)	
Neither Agree Or Disagree	3 (11.1%)	0 (0.0%)	0 (0.0%)	3 (7.9%)	
Strongly Agree	4 (14.8%)	3 (50.0%)	3 (60.0%)	10 (26.3%)	
**Knowing how my body may respond to certain prescription drugs would be useful to me immediately**					0.763[1]
N-Miss	2	0	1	3	
Agree	18 (69.2%)	3 (50.0%)	4 (80.0%)	25 (67.6%)	
Disagree	1 (3.8%)	0 (0.0%)	0 (0.0%)	1 (2.7%)	
Neither Agree Or Disagree	4 (15.4%)	1 (16.7%)	1 (20.0%)	6 (16.2%)	
Strongly Agree	3 (11.5%)	2 (33.3%)	0 (0.0%)	5 (13.5%)	
**Knowing how my body may respond to certain prescription drugs would give me more control over my health**					0.570[1]
N-Miss	1	0	1	2	
Agree	17 (63.0%)	2 (33.3%)	2 (40.0%)	21 (55.3%)	
Disagree	1 (3.7%)	0 (0.0%)	0 (0.0%)	1 (2.6%)	
Neither Agree Or Disagree	4 (14.8%)	1 (16.7%)	1 (20.0%)	6 (15.8%)	
Strongly Agree	4 (14.8%)	3 (50.0%)	2 (40.0%)	9 (23.7%)	
Strongly Disagree	1 (3.7%)	0 (0.0%)	0 (0.0%)	1 (2.6%)	
**Knowing how my body may respond to certain prescription drugs would be useful to me in the future**					0.118[1]
N-Miss	1	0	1	2	
Agree	18 (66.7%)	2 (33.3%)	1 (20.0%)	21 (55.3%)	

Neither Agree Or Disagree	2 (7.4%)	0 (0.0%)	1 (20.0%)	3 (7.9%)	
Strongly Agree	7 (25.9%)	4 (66.7%)	3 (60.0%)	14 (36.8%)	
**How valuable are your pharmacogenomics results?**					1.000[1]
N-Miss	2	0	1	3	
Extremely Valuable	5 (19.2%)	1 (16.7%)	1 (20.0%)	7 (18.9%)	
Not At All Valuable	1 (3.8%)	0 (0.0%)	0 (0.0%)	1 (2.7%)	
Quite Valuable	12 (46.2%)	3 (50.0%)	2 (40.0%)	17 (45.9%)	
Slightly Valuable	8 (30.8%)	2 (33.3%)	2 (40.0%)	12 (32.4%)	
**Would you recommend you friends or family to have pharmacogenomics testing?**					0.277[1]
N-Miss	1	0	0	1	
Don't Know	6 (22.2%)	3 (50.0%)	0 (0.0%)	9 (23.1%)	
No	2 (7.4%)	0 (0.0%)	0 (0.0%)	2 (5.1%)	
Yes	19 (70.4%)	3 (50.0%)	6 (100.0%)	28 (71.8%)	

1.Fisher's Exact Test for Count Data
